# FcRY is a key molecule controlling maternal blood IgY transfer to yolks during egg development in avian species

**DOI:** 10.3389/fimmu.2024.1305587

**Published:** 2024-02-29

**Authors:** Mayuko Okamoto, Ryo Sasaki, Koki Ikeda, Kasumi Doi, Fumiya Tatsumi, Kenzi Oshima, Takaaki Kojima, Shusei Mizushima, Keisuke Ikegami, Takashi Yoshimura, Kyohei Furukawa, Misato Kobayashi, Fumihiko Horio, Atsushi Murai

**Affiliations:** ^1^ Laboratory of Animal Nutrition, Graduate School of Bioagricultural Sciences, Nagoya University, Nagoya, Japan; ^2^ Laboratory of Molecular Bioregulation, Graduate School of Bioagricultural Sciences, Nagoya University, Nagoya, Japan; ^3^ Laboratory of Molecular Biotechnology, Graduate School of Bioagricultural Sciences, Nagoya University, Nagoya, Japan; ^4^ Department of Biological Sciences, Faculty of Science, Hokkaido University, Sapporo, Japan; ^5^ Laboratory of Animal Integrative Physiology, Graduate School of Bioagricultural Sciences, Nagoya University, Nagoya, Japan; ^6^ Institute of Transformative Bio-Molecules (WPI-ITbM), Nagoya University, Nagoya, Japan

**Keywords:** maternal immunity, avian species, immunoglobulin, IgY, Fc receptor, egg yolk, FcRY

## Abstract

Maternal immunoglobulin transfer plays a key role in conferring passive immunity to neonates. Maternal blood immunoglobulin Y (IgY) in avian species is transported to newly-hatched chicks in two steps: 1) IgY is transported from the maternal circulation to the yolk of maturing oocytes, 2) the IgY deposited in yolk is transported to the circulation of the embryo via the yolk sac membrane. An IgY-Fc receptor, FcRY, is involved in the second step, but the mechanism of the first step is still unclear. We determined whether FcRY was also the basis for maternal blood IgY transfer to the yolk in the first step during egg development. Immunohistochemistry revealed that FcRY was expressed in the capillary endothelial cells in the internal theca layer of the ovarian follicle. Substitution of the amino acid residue in Fc region of IgY substantially changed the transport efficiency of IgY into egg yolks when intravenously-injected into laying quail; the G365A mutant had a high transport efficiency, but the Y363A mutant lacked transport ability. Binding analyses of IgY mutants to FcRY indicated that the mutant with a high transport efficiency (G365A) had a strong binding activity to FcRY; the mutants with a low transport efficiency (G365D, N408A) had a weak binding activity to FcRY. One exception, the Y363A mutant had a remarkably strong binding affinity to FcRY, with a small dissociation rate. The injection of neutralizing FcRY antibodies in laying quail markedly reduced IgY uptake into egg yolks. The neutralization also showed that FcRY was engaged in prolongation of half-life of IgY in the blood; FcRY is therefore a multifunctional receptor that controls avian immunity. The pattern of the transport of the IgY mutants from the maternal blood to the egg yolk was found to be identical to that from the fertilized egg yolk to the newly-hatched chick blood circulation, via the yolk sac membrane. FcRY is therefore a critical IgY receptor that regulates the IgY uptake from the maternal blood circulation into the yolk of avian species, further indicating that the two steps of maternal–newly-hatched IgY transfer are controlled by a single receptor.

## Introduction

Maternal immunity, a passive immunoglobulin (Ig) transfer, plays a key role in neonates to prevent infections during the developmental stages of their immune systems. In birds, IgY, functionally homologous to mammalian IgG, is transported from the hen to the newly-hatched chick in two steps (1–3); in the first step, maternal IgY is transported to the yolk of developing oocytes, while in the second step, the deposited IgY is transported from the yolk to the circulation of the embryo via the yolk sac membrane during late embryonic development. The second step relies on an IgY-Fc receptor, FcRY, the functional homolog of the mammalian neonatal Fc receptor, FcRn (4, 5). During the first step, IgY is believed to be selectively transported into the oocytes by receptor-mediated uptake. However, the receptor responsible for maternal blood IgY transfer into yolks has not been identified.

A study on the structural requirements of IgY for egg yolk transport is vital to identify the receptor responsible for IgY transfer in the first step. Intravenously-injected IgY and its Fc fragments are incorporated into egg yolks of laying hens more than Fab and F(ab’) fragments (6). Additionally, IgY-Fc mutants with an amino acid residue substituted at the interface of the Cυ3–Cυ4 domain produce large differences in uptake into the egg yolk (7, 8). These results indicate that a receptor, which recognizes specific regions of the IgY-Fc domain, is involved in the first step of maternal IgY transfer.

FcRY was isolated from the chicken yolk sac membrane as the receptor responsible for the second step (4, 9). FcRY is a member of the mannose receptor family, with 180 kDa as its transmembrane form (10). FcRY has 55% homology to mammalian (human) phospholipase A_2_ receptor, but the function is similar to mammalian FcRn. FcRY has IgY-Fc-binding capacity due to a conformational change at ~pH 6.0 and releases IgY above pH 7.4 (11). An experiment using FcRY-expressing cell lines has shown that FcRY transcytoses IgY from the apical to the basolateral site (12). Until now, it has been proposed that FcRY is not involved in the first step of maternal IgY transfer (13, 14) from observations of human IgG (hIgG) transport into avian egg yolks. Human IgG is believed to be transported into egg yolks by the same mechanism of avian IgY, but hIgG does not bind to FcRY (4). However, nobody has examined whether hIgG presents the same transport ability as IgY. IgY-deficiency in bursectomized chickens displayed an enhanced ability to transport IgY into the egg yolks and microarray analysis revealed an increased expression of FcRY in the ovarian follicles (15). These results suggest that FcRY may be the receptor responsible for IgY transport in the first, as well as the second step.

The present study investigated the hypothesis that FcRY was a key molecule in regulating IgY transport from the maternal circulation to the egg yolk. This is the first direct comparison of yolk-transport amounts of IgY and hIgG and includes a detailed localization of FcRY in ovarian follicles of chickens and quail. To clarify whether the IgY/FcRY interaction produces the yolk-transport amounts in the first step, the relationship between the binding properties of IgY-Fc mutants/FcRY and the transport ability of the IgY-Fc mutants into the yolk was examined. Finally, *in vivo* FcRY function in maternal IgY transfer was examined by neutralizing FcRY-binding ability to IgY.

## Materials and methods

### Reagents and conventions

All chemicals were of analytical grade. IgY amino acid residues were numbered according to the system of Suzuki and Lee (16).

### Recombinant proteins

An expression vector containing chicken FcRY, lacking the membrane-spanning domain (the residues at 36–1396) from the template vector (donated by Dr. Pamela J. Bjorkman, California Institute of Technology), named secretory FcRY, was constructed.

The cDNA encoding the quail IgY (qIgY) υ-heavy chain was isolated from a quail splenocyte cDNA library (8). The gene encoding the Fc regions (H chain residues at 231–568) was isolated by polymerase chain reaction (PCR) from the template cDNA, and the PCR product was ligated into the *Kpn*I-*Xho*I cloning site of pSecTag2, a mammalian expression vector (Invitrogen, Carlsbad, CA), with a C-terminal 6 × His tag. The constructed expression vector was used to synthesize recombinant quail wild-type IgY-Fc (designated WT). The IgY-Fc mutants (Y363A, G365A, G365D, G365S, and N408A) were generated using QuickChange II Mutagenesis Kit (Stratagene, Santa Clara, CA) or the KOD -Plus- Mutagenesis Kit (Toyobo, Osaka, Japan) according to the manufacturer’s instructions. Another IgY-Fc and its mutants, lacking the His tag, were also generated by inserting a stop codon in front of the His tag codon. The sequences of the mutant genes were verified by an ABI3130 sequencer (Applied Biosystems, Foster City, CA) or by Eurofins Genomics Inc. (Tokyo, Japan).

The generated secretory FcRY and IgY-Fc constructs were then transiently transfected into CHO-S cells using FreeStyle™ MAX reagent (Invitrogen) or the ExpiCHO™ Expression System (Thermo Fisher Scientific, Waltham, MA), following standard protocols. The expressed proteins were purified by His Spin Trap affinity columns, according to the manufacturer’s instructions (GE Healthcare, Waukesha, WI). The IgY-Fc lacking the His tag was purified using HiTrap™ NHS-activated HP columns (GE Healthcare), immobilized with rabbit anti-qIgY (17). The purification and molecular mass of the secretory FcRY and IgY-Fc were analyzed using SDS-PAGE under reducing and non-reducing conditions. Gels were stained with 0.5% Coomassie Brilliant Blue R-250 (CBB). The concentrations of the proteins were determined by measuring absorption at 280 nm (*A*_280_), with 1.4 as the extinction coefficient (*A*_280_/1.4 = concentration in mg/mL). Following the analyses, the formations of standard curve were checked by an original enzyme-linked immunosorbent assay (ELISA) system detecting qIgY (17).

The purified proteins were labeled with digoxigenin (DIG) using a DIG protein labeling kit (Roche Diagnostics, Indianapolis, IN) as necessary, according to the recommendations of the manufacturer.

### Antibodies against FcRY

To obtain a specific antibody against FcRY, the purified secretory chicken FcRY was used as an antigen. The rabbits were immunized by Eurofins Genomics Inc. The acquired blood serum was mixed with 3 M ammonium sulfate for the precipitation of antibodies. The pellets of antibodies were resuspended in Tris-buffered saline (TBS) and dialyzed overnight against TBS at 4°C.

For the isolation of the FcRY-specific antibody, affinity chromatography consisting of a HiTrap™ NHS-activated HP column immobilized with the secretory chicken FcRY was used. The acquired antibodies were dialyzed against phosphate-buffered saline (PBS) (pH 7.4), and they were used for both FcRY detection and neutralizing FcRY binding activity.

### Experimental birds and their management

Commercial female Japanese quail (*Coturnix japonica*) and White Leghorn-type commercial chickens (*Gallus gallus*; Julia Light^®^) were purchased from a local hatchery (Cyubu-kagaku-shizai, Nagoya, Japan). Female quail of a closed colony strain (WE, White Egg shell) was supplied by the National BioResource Project - Chicken and Quail, Nagoya University (Nagoya, Japan). All birds were maintained individually with free access to water and a commercial diet (Quail: Uzura-super^®^; Toyohashi Feed Mills, Toyohashi, Japan, Chicken: S-seven; Nosan Co., Kanagawa, Japan). The photoperiod was set at 16L:8D during the experiment. The room temperatures were controlled at 24 ± 2°C. Ten- to 30-week-old quail and 64-week-old chickens laying consistently were used for the experiments and their egg production was recorded daily. The animal care was in total compliance with the guidelines of the Nagoya University Policy on Animal Care and Use (2012030901, 2019031204, AGR2019044, A210510, A220021).

### Intravenous injection of Igs and IgY-Fc mutants to laying chicken and quails and preparation of egg yolk extract

The quail laying regularly were injected intravenously with 20 µg/100 g BW of DIG-labeled chicken IgY (cIgY; Rockland, Philadelphia, PA), DIG-labeled hIgG (Sigma-Aldrich, St. Louis, MO), DIG-labeled IgY-Fc, and DIG-labeled IgY-Fc mutants dissolved in PBS (20 μg Igs/200 μL). Each chicken laying regularly was also injected intravenously with 100 µg/1,500 g BW of DIG-labeled cIgY or DIG-labeled hIgG dissolved in PBS (100 μg Igs/200 μL). All laid eggs were collected for 6 or 7 days after the injection and stored at 4°C until analysis. The egg yolk was separated from egg white to measure the incorporated proteins. Egg yolk extracts containing IgY and the incorporated proteins were prepared as described previously (7). The final solution was used for the determination of the incorporated protein concentrations using ELISA to detect conjugated DIG. Blood samples were collected 3 h post-injection. The concentration of the injected Igs in serum was determined using ELISA.

### In ovo injection of IgY-Fc mutants

At day zero of incubation, a small hole was drilled in the eggshell of fertilized eggs (Japanese quail) using a 27G needle. An amount of 150 μL of albumen from the eggs was discarded. Following this, the DIG-labeled IgY-Fc mutants, WT, Y363A, G365A, and hIgG at 17 μg diluted in 25 μL saline with 1% (v/v) 100 × penicillin–streptomycin solution (FUJIFILM Wako, Osaka, Japan) were injected into the egg yolks. After the injection, 20 μL of 100 × penicillin–streptomycin solution (diluted in PBS) was injected into the albumen of the eggs. The hole was sealed with scotch tape. The eggs were incubated at 37.6°C with 55 to 60% relative humidity and turned once per 1 h until day 15 of incubation. Immediately after hatching at day 18, chicks were intraperitoneally administered with anesthetic agents (0.75 μg/kg BW Medetomidine hydrochloride, 4 μg/kg BW Midazolam, 5 μg/kg BW Butorphanol tartrate) diluted in 80 μL of saline. Blood samples were collected from the heart. The concentrations of injected Igs in the serum were determined using ELISA.

### Injection of neutralizing antibodies in laying quail

To neutralize FcRY binding activity, each regularly-laying quail was injected intravenously with 1 mg/100 g BW of FcRY-specific antibody and 20 µg/100 g BW of DIG-labeled WT for the tracer antibody, diluted in 500 μL of saline. All laid eggs were collected for 5 days after the injection and stored at 4°C until analysis. Blood samples were collected at 1- and 3-h post-injection. The concentrations of the injected DIG-labeled WT and endogenous qIgY in serum were determined using ELISA.

### ELISA

The concentration of native cIgY in the egg yolk extracts was measured using a commercial Chicken IgG ELISA quantitation kit (Bethyl Laboratories, Montgomery, TX), according to the manufacturer’s instructions. The concentrations of native qIgY in blood samples were measured using an original ELISA (17). The concentrations of DIG-labeled Igs in the egg yolk extracts and in blood samples were measured using an original ELISA detecting conjugated DIG (18).

### Western blotting analysis

The expression pattern of FcRY was examined using western blotting analysis. Each tissue sample from the chickens [white ovarian follicle, theca layer, and granulosa cell (GC) layer of yellow ovarian follicle] was homogenized with lysis buffer. The egg yolk and tissue homogenate samples (equivalent to 2 or 5 µg of protein) were separated using SDS-PAGE under reducing conditions. The blotting PVDF membranes were first incubated with rabbit anti-chicken FcRY antibody (1:2,500) or rabbit anti-human GAPDH (1:1,000; Santa Cruz Biotechnology, Dallas, TX) and then with HRP-conjugated goat anti-rabbit IgG antibody (1:2,000; Cell Signaling technology, Danvers, MA). The membranes were visualized using a chemiluminescence detection method (SuperSignal^®^ West Dura Extended Duration Substrate; Thermo Fisher Scientific) with a charge-coupled device camera (AE-6960/C; Atto, Tokyo, Japan).

### Visualization of FcRY in ovarian follicles

The FcRY expression in ovarian follicles was visualized using immunohistochemistry and immunofluorescence detection. The chickens were anesthetized with pentobarbital, and perfused intracardially with heparinized physiological saline for 5 min. The ovarian follicle (F3) from the chicken was then fixed with Mildform^®^ (FUJIFILM Wako). The quail were euthanized by decapitation. The ovarian follicle (F4) was removed and fixed with Mildform^®^. All samples were embedded in paraffin.

The paraffin-embedded samples were sliced into 3-μm sections using a microtome. The sections were incubated overnight with rabbit anti-chicken FcRY antibody (1:500 in blocking buffer) at 4°C. Similar concentrations of serum-derived rabbit IgG (FUJIFILM Wako) were used for isotype control. In absorption control, 50 μL of secretory FcRY (5 μg/mL) was added to the anti-chicken FcRY primary antibody and incubated overnight at 4°C before adding to the sections. After washing, the sections were incubated with biotin-conjugated goat anti-rabbit IgG and then with avidin-biotinylated HRP complex (PK4001; Vector Laboratories, Newark, CA). Finally, the sections were visualized with diaminobenzidine solution (Dako, Glostrup, Denmark) followed by counterstaining with hematoxylin. The sections were observed under an upright microscope (BX51; Olympus, Tokyo, Japan) equipped with a charge-coupled device camera (DP20; Olympus) and microscope software (DP2-BSW ver. 2.2; Olympus). Images were captured at 40 × objective lens or at 100 × oil-immersion objective lens.

Using immunofluorescence detection, the samples were sliced into 5-μm sections. To observe FcRY, the sections were treated overnight with rabbit anti-chicken FcRY antibody (1:125) at 4°C. After washing, the sections were incubated with biotin-conjugated goat anti-rabbit IgG (BA-1000; Vector Laboratories, 1:250) in blocking buffer for 1 h in a dark room. The sections were then washed and incubated with Dylight^®^488-conjugated streptavidin (SA-5488; Vector Laboratories, 1:100 in PBS) for 30 min in a dark room. To observe the vascular endothelial cells of quail, a quail vascular endothelial cell marker, QH1 (AB_531829; DSHB, 1:125) and AlexaFluor^®^ 568-conjugated anti-mouse IgG (ab175473; Abcam, Cambridge, UK, 1:1,000) were used as the primary and secondary antibodies, respectively. Subsequently, all sections were quenched and DAPI-stained using the Vector^®^ TrueView^®^ Autofluorescence Quenching Kit with DAPI (SP-8500; Vector Laboratories) for 5 min in a dark room. The sections were sealed and examined with a confocal laser scanning microscope (FV1000-D; Olympus) equipped with operational software (FV10-ASW). Images were captured at 100 × oil-immersion objective lens. The acquired images were adjusted for contrast using Fiji ImageJ ver. 1.53c (19). The outlines of cells in the ovarian follicle were traced on the basis of the differential interference contrast image and the fluorescence signal.

### Visualization of IgY-Fc mutants in ovarian follicles

Deposition and infiltration of the injected WT and Y363A mutants into ovarian follicles were visualized using a standard avidin-biotinylated HRP complex method. DIG-labeled WT and Y363A mutants (400 µg) were injected intravenously into laying quail. After 0.5 h, the quail were euthanized by decapitation and the ovarian follicles were removed and fixed with Mildform^®^. The third largest ovarian follicle (F3) was paraffin-embedded, and the samples were sliced with a microtome into 5-µm sections. To detect the perivitelline layer, the sections were incubated overnight with rabbit anti-qZP3 IgG (1:200; donated by Dr. Tomohiro Sasanami, Shizuoka University, Japan) in a blocking buffer at 4°C and then with tetra methyl rhodamine isothiocyanate (TRITC)-conjugated goat anti-rabbit IgG in a blocking buffer for 1 h. To examine the deposition of IgY-Fc mutants, they were incubated overnight with monoclonal anti-DIG mouse IgG (1:700) at 4°C after the second blocking step, and then treated with biotinylated anti-mouse IgG1 (1:400). They were then treated with Fluorescein Avidin DCS (1:250) for 1 h. Finally, the sections were loaded with fluorescent mounting medium and observed using a fluorescence microscope (BZ-X; Keyence, Osaka, Japan).

### IgY-binding activities against FcRY

Copper-coated 96 well plates (Pierce™ Copper Coated High-Capacity Plates, Thermo Fisher Scientific) were incubated with 100 μL/well of the secretory FcRY with His tag (10 μg/mL) in PBS with 0.05% Tween 20 and 20 mM MES/HEPES (pH 4.0–7.4) for 60 min at room temperature. The plates were washed and loaded with 150 μL/well of blocking solution (pH 4.0–7.4) containing 1% (v/v) bovine serum albumin (Sigma-Aldrich) for 30 min. After washing, the plates were incubated with the native cIgY, qIgY, or IgY-Fc mutants (1.1-1,111 nM, pH 4.0–7.4) at 100 μL/well for 60 min. In the competitive binding assay, the plates were incubated with the DIG-labeled qIgY (27.8 nM final concentration) and subsequently loaded with several concentrations of IgY-Fc mutants as a competitor at pH 6.0 for 90 min. After washing, a basic solution (pH 8.0) was added to liberate bound Igs from the FcRY. The concentrations of Igs in the collected solutions were determined using ELISA.

To assess the ability of the FcRY-specific antibody to neutralize IgY binding activity, graded amounts of FcRY-specific antibody and native cIgY (111.1 nM final concentration) were reacted with the plates immobilized with FcRY; IgY bound to FcRY was then determined using ELISA.

### Bio-layer interferometry

Binding of Igs to biosensor surfaces was evaluated using BLI on BLItz™ (45–5000; Sartorius, Goettingen, Germany) in advanced kinetics mode, following the manufacture’s protocol. PBS buffer (0.05% Tween 20 and 20 mM MES at pH 5.5 or 20 mM HEPES at pH 7.4) was used in initial baseline step, and PBS buffer supplemented with 0.5% BSA was used in the later steps. All solutions were filtered using 0.2-μm syringe filters. Before measurements, Ni-NTA sensor chips (Sartorius) were hydrated using PBS (20 mM MES at pH 5.5 or 20 mM HEPES at pH 7.4) for 10 min. Chicken FcRY (400 nM) was immobilized on the sensor chip via a His tag in the loading step (180 s). After the baseline step, the sensor chips were dipped into antibody solutions (50–600 nM) in the association step (120 s). The sensor chips were subsequently soaked with dissociation buffer (120 s). For each subsequent run, a new biosensor was prepared (biosensors were not re-used). Data analysis and fitting (1:1) were achieved using BLItz Pro Software 1.0.

### Statistical analyses

The data were analyzed using one-way analysis of variance (ANOVA). The mean values were compared using Turkey–Kramer’s test or Dunnett’s test. Comparisons between two groups were performed using unpaired-Student’s *t*-tests. All error bars are expressed as the mean ± standard deviation (SD), and differences between means were considered to be significant at *p* < 0.05. Statistical analysis was performed with BellCurve for Excel (ver. 3.21) or GraphPad Prism 9.4.0 (GraphPad Software, Inc., La Jolla, CA, USA).

## Results

### Comparison of human IgG and avian IgY uptake into egg yolks

**Figure 1:**
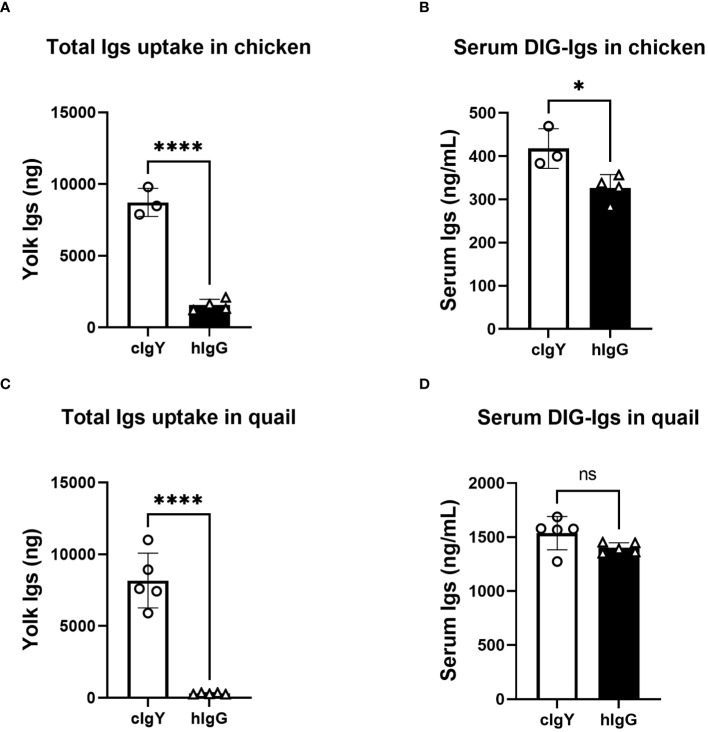
Uptake of exogenously-injected avian IgY into egg yolks is markedly higher that of human IgG. **(A, B)** Chickens were intravenously injected with 100 μg/1,500 g BW of DIG-labeled cIgY or DIG-labeled hIgG dissolved in PBS (n = 3 or 4). All eggs laid were collected for 7 d after the injection and uptake into the egg yolks was measured using ELISA **(A)**. The blood samples were collected 3 h after injections and concentrations in the serum were measured **(B)**. **(C, D)** Quail were intravenously injected with 20 μg/100 g BW of DIG-labeled cIgY or DIG-labeled hIgG dissolved in PBS (n = 5). All eggs laid were collected for 7 d after the injection and total uptake into the egg yolks was measured **(C)**. The blood samples were collected 3 h after injections and concentrations in the serum were measured **(D)**. Vertical bar indicates mean ± standard deviation (SD). Statistically significant p values were determined using unpaired Student’s *t*-tests: *p < 0.05, ****p < 0.0001.

If FcRY is the receptor responsible for blood IgY transfer into yolks, uptake of blood hIgG into yolks must be extremely low compared to that of avian IgY. To test this, the uptake of intravenously injected DIG-labeled hIgG or DIG-labeled cIgY into egg yolks was compared. Because the uptake of injected cIgY into egg yolks was comparable to that of injected qIgY in quail (18), cIgY was used here as a tracer. In chickens and quail, the uptake of cIgY into egg yolks were markedly higher than that of hIgG (**Figures 1A, C**). Only small or statistically insignificant differences were observed in blood concentrations of cIgY and hIgG collected 3 h after the injection (**Figures 1B, D**), suggesting small or no influence of blood clearance on their uptake. These results suggest a possibility that FcRY facilitates IgY-specific transfer into egg yolks.

### Avian FcRY is expressed in capillary endothelial cells of the ovarian follicle

**Figure 2:**
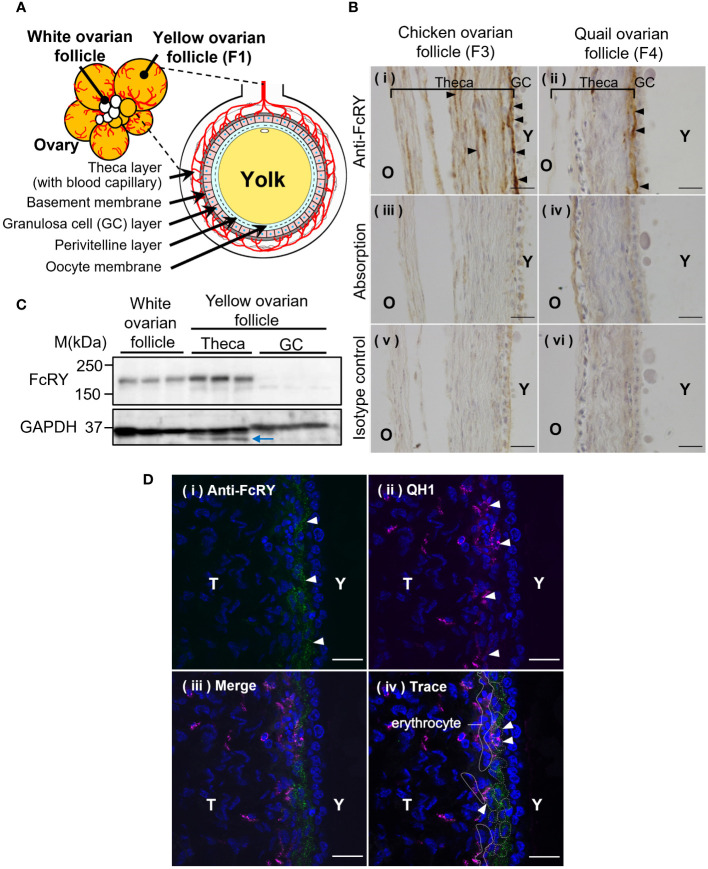
Avian FcRY is expressed in the capillary endothelial cells of ovarian follicles. **(A)** Graphical illustration of the ovarian follicle of laying birds. **(B)** Detection of FcRY in a chicken ovarian follicle (F3) and a quail ovarian follicle (F4) using immunohistochemical analysis (× 100). The sections were stained with anti-FcRY antibody. In the absorption control, the anti-FcRY antibody was incubated with recombinant secretory FcRY overnight at 4°C before adding it to the sections. Scale bars, 20 μm. **(C)** Detection of FcRY in the chicken yolk sac, white (small), and yellow (large) ovarian follicles using western blotting analysis (n = 3). Multiple surface layers of yellow ovarian follicles were separated into the theca layer and the GC layer. The blue arrow indicates the non-specific binding bands. **(D)** Confocal images of quail ovarian follicle stained with anti-FcRY antibody (green) and with QH1, a quail endothelial cell marker (magenta; original magnification, ×100). The nuclei were stained with DAPI (blue). Scale bars, 20 μm. GC, granulosa cell; T, theca layer; Y, yolk.

Immunohistochemical analysis showed that FcRY was expressed in a variety of tissues, including ovarian follicles (**Figure 2B**), the liver, kidney, spleen, jejunum, and the embryonic yolk sac membrane (**Supplementary Figure 1**, **Supplementary Table 1**). In both chicken and quail ovarian follicles, FcRY signals were detected in the internal theca layers, close to the GC layer (**Figures 2A** **, B**-i, ii). Absorption of the FcRY antibody by recombinant FcRY completely eliminated these signals to the same level as the isotype control (**Figure 2B**-iii-vi). Western blotting analysis showed that FcRY protein, with a molecular mass of 180 kDa, was expressed in both the developing white ovarian and the maturing yellow ovarian follicles (**Figure 2C**). Similar to the immunohistochemical analysis, FcRY was expressed in the theca layer but not in the GC layer.

The ends of the blood capillaries in the internal theca layer of the ovarian follicle are distributed throughout the surrounding region, close to the basement membrane (**Figure 2A**). It was hypothesized that FcRY in the theca layer of ovarian follicles would be expressed at the terminal capillaries and transport IgY to the internal layers of the ovarian follicles. Confocal microscopy analysis indicated an FcRY signal in a line near the basement membrane of the theca layer (**Figure 2D**-i; green). The QH1 signal, a quail endothelial cell marker, was detected throughout the theca layer, but intensively in the internal theca layer (**Figure 2D**-ii; magenta). The FcRY signals were colocalized with QH1 (**Figure 2D**-iii, iv), suggesting that FcRY was present in the vascular endothelial cells.

### Transport efficiency of IgY-Fc mutants into egg yolks and newly-hatched chicks

Recombinant IgY-Fc and its mutants can be used to clarify whether the IgY/FcRY interaction controls maternal IgY transfer. IgY-Fc mutants substituting one amino acid residue for alanine were generated in a previous study and have variable abilities for transport into yolks when injected into laying birds (8). Analysis of the transport efficiency and binding properties of IgY-Fc mutants can elucidate the involvement of FcRY in maternal IgY transfer.

**Figure 3:**
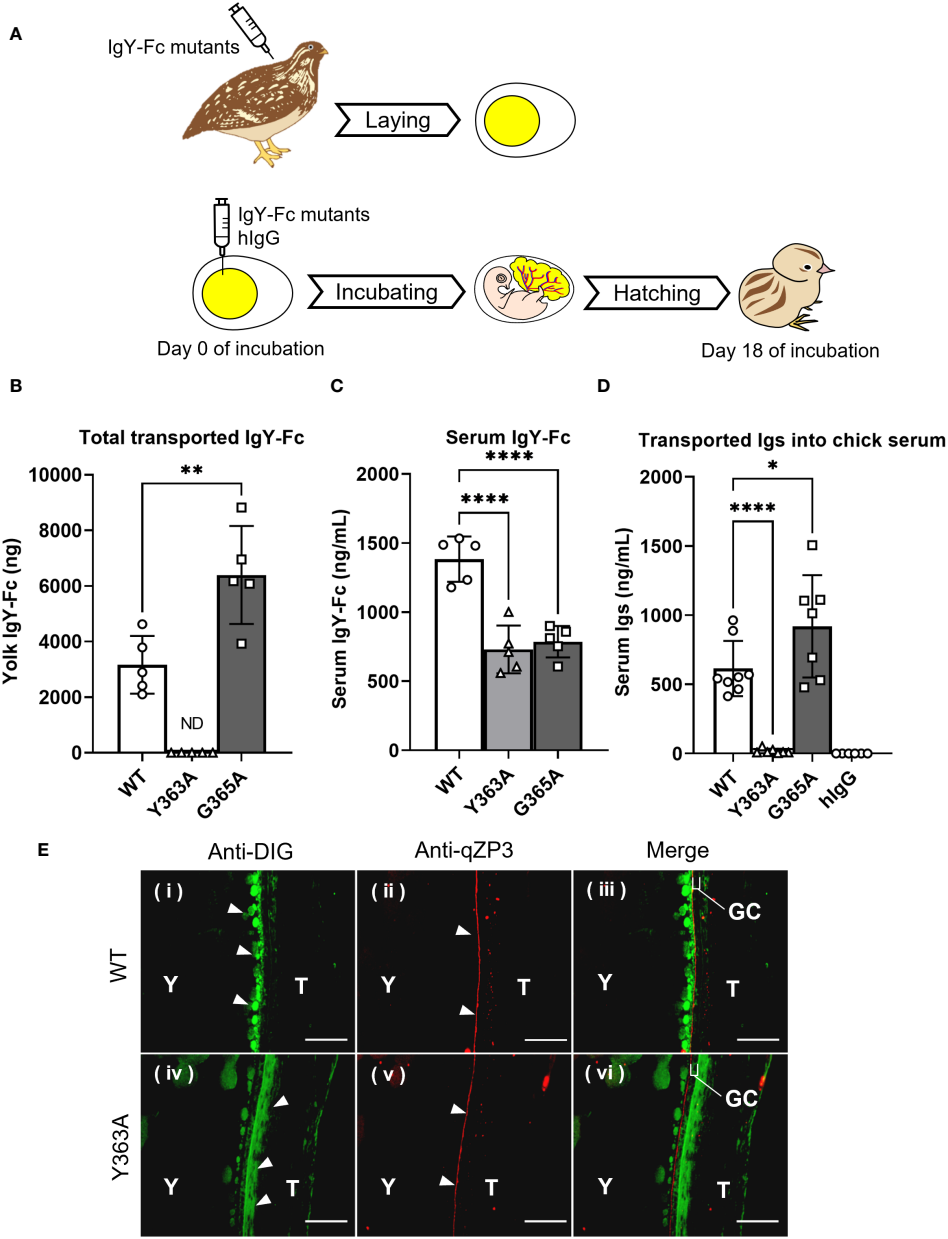
Variable uptakes of IgY-Fc mutants with a single amino acid substitution into egg yolks and newly-hatched chicks. **(A)** Schematic representation of the experimental approach from injection to sampling. **(B, C)** Quail were intravenously injected with 20 μg/100 g BW of DIG-labeled recombinant quail IgY-Fc (WT, Y363A, G365A; n = 5). All laid eggs were collected for 6 d after the injection and the total uptake into the egg yolks was measured using ELISA **(B)**. The blood samples were collected 3 h after injections and concentrations in the serum were measured **(C)**. **(D)** The egg yolks of fertilized quail eggs were injected with 17 μg of DIG-labeled quail IgY-Fc (WT, Y363A, G365A) and DIG-labeled hIgG (n = 6–9). After hatching, the blood samples of newly-hatched chicks were collected, and the concentrations of injected Igs in each sample were detected using ELISA. Vertical bar indicates mean ± standard deviation. Statistically significant p-values were determined using unpaired Student’s *t*-tests in **(B)** or analysis of variance with Dunnett’s multiple comparisons in **(C, D)**, *p < 0.05, **p < 0.01, ****p < 0.0001. **(E)** Quail were intravenously injected with 400 μg of DIG-labeled quail WT or Y363A, and the third largest ovarian follicles were collected 0.5 hour after injection. The sections were prepared from the ovarian follicles, and then they were incubated with anti-DIG antibody (green) to detect the WT or Y363A. The perivitelline membrane was detected using anti-qZP3 (red; original magnification, ×40). Scale bars, 40 μm. GC, granulosa cell layer; T, theca layer; WT, recombinant quail wild-type IgY-Fc; Y, yolk.

The uptake of the injected quail IgY-Fc mutants into quail egg yolks was analyzed during the first step (**Figure 3A** top): G365A was detected at two-fold higher concentrations in the egg yolks compared to WT, whereas Y363A was undetected (**Figure 3B**); the blood concentration of WT was slightly higher than that of G365A and Y363A (**Figure 3C**). These results indicate that the blood clearance of the mutants did not account for the differences in their uptake into the egg yolk.

To examine whether the transport pattern of the mutants in the first step was identical to the pattern in the second step, the IgY-Fc mutants and hIgG were injected into yolks of fertilized quail eggs and incubated, followed by measurement of the transferred mutants in blood of newly-hatched chicks (**Figure 3A** bottom). The G365A concentration in blood was 1.5-fold higher than that of the WT, whereas the Y363A concentration was less than 3% of the WT; hIgG was undetectable (**Figure 3D**).

Overall, the transport patterns of the IgY-Fc mutants and hIgG from the yolk sac to the embryonic blood circulation was consistent with that of the injected IgY-Fc mutants and hIgG into the quail egg yolks, suggesting that the same receptor may contribute to both IgY transport in the first and second steps.

Localization of the injected DIG-labeled WT and Y363A in quail ovarian follicles was visualized using a fluorescent microscope. WT (green) accumulated in the yolks of oocytes on the inside of the qZP3 signal (marker of perivitelline layer, red; **Figure 3E**-i–iii). In contrast, Y363A was detected outside of the qZP3 signal and GC layer (**Figure 3E**-iv–vi). These results suggest that there is a key molecule regulating the uptakes of IgY-Fc and its mutants in the internal theca layer of the ovarian follicle, where FcRY is localized.

### Binding activities of IgY-Fc mutants to FcRY immobilized in the 96-well plate

**Figure 4:**
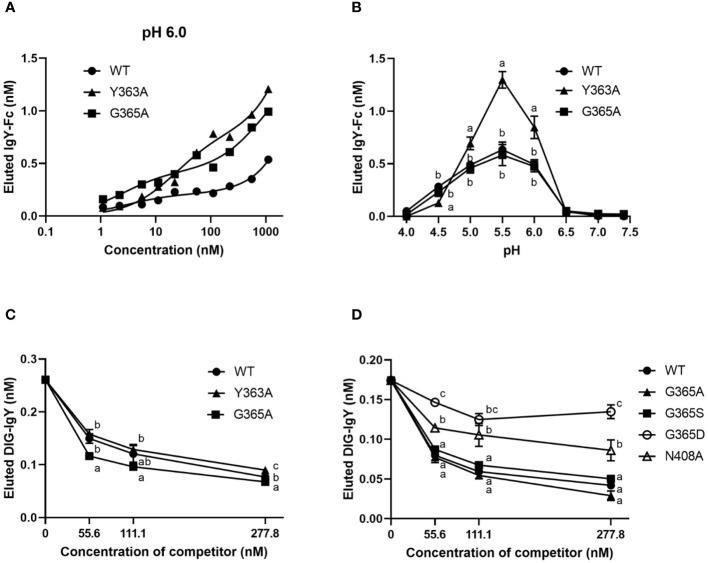
The binding activity of IgY-Fc mutants to FcRY is consistent with the transport of these mutants to egg yolks. **(A)** Copper-coated 96 well plates were incubated with the secretory FcRY (10 μg/mL). The plates were then incubated with the quail IgY-Fc mutants at pH 6.0 (1.1–1,111 nM; n = 1). After washing, a basic solution of pH 8.0 was added to liberate the bound mutants from the FcRY. The concentrations of IgY-Fc mutants in the collected solutions were determined using ELISA. **(B)** pH-dependent binding activity of quail IgY-Fc mutants (55.6 nM) to FcRY at pH 4.0–7.4 (n = 3). **(C, D)** DIG-labeled chicken IgY (27.8 nM final concentration) was incubated with an FcRY-immobilized plate with the graded concentrations of IgY-Fc mutants as a competitor at pH 6.0 (n = 3). The concentration of the DIG-IgY bound to FcRY was detected using ELISA. The results are shown as mean ± standard deviation. The letters a, b, and c within the same concentration represent significant differences at *p* < 0.05, as determined using analysis of variance with Turkey–Kramer’s multiple comparison test. WT, recombinant quail wild-type IgY-Fc.

We hypothesized that the binding activity of IgY-Fc and its mutants to FcRY controls their uptake into egg yolks. FcRY is known to bind to IgY below pH 6.0 and dissociate above pH 7.4 (4). Consistent with previous studies, at pH 6.0, both chicken and quail IgYs bonded to FcRY immobilized in the plate in a dose-dependent manner but failed to bind at all at pH 7.4 (**Supplementary Figure 2**). All the IgY-Fc mutants also bonded to FcRY in a dose-dependent manner when incubated at pH 6.0 (**Figure 4A**). Compared to WT, G365A bonded more avidly to FcRY; Y363A bonded more strongly to FcRY than WT and G365A, while none of the mutants bonded at pH 7.4 (**Supplementary Figure 3A**). Further examination of the binding to FcRY at graded pH (pH 4.0–7.4) showed that all mutants bonded most strongly to FcRY at pH 5.5 (**Figure 4B**). Approximately twice as much Y363A was found to bind strongly at pH 5.0–6.0 than WT and G365A.

The competitive ability of each mutant was compared using DIG-labeled IgY. All mutants inhibited DIG-IgY binding to FcRY in a dose-dependent manner (**Figures 4C, D**). Y363A showed a similar inhibition activity to WT, whereas G365A more effectively inhibited the DIG-IgY binding to FcRY than WT. G365S, which has similar ability to transport IgY into egg yolks as G365A (8), also strongly inhibited the binding of DIG-IgY (**Figure 4D**). G365D and N408A, which are not usually transported to egg yolks (8), were substantially less potent in inhibiting DIG-IgY binding than the WT. These results indicate that the binding activity of IgY-Fc mutants to FcRY was consistent with the transport pattern of these mutants to egg yolks.

### Binding affinities and kinetic parameters of IgY-Fc mutants to FcRY using the BLI system

**Figure 5:**
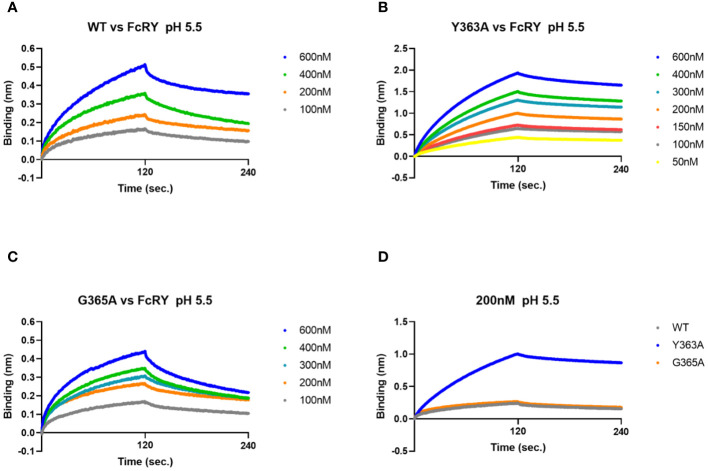
The G365A mutant with a strong efficiency of transport to the egg yolk has a higher binding affinity to FcRY than to the WT. **(A–C)** FcRY (400 nM) was immobilized on the sensor chip. The sensor chips were dipped into WT **(A)**, Y363A **(B)**, or G365A **(C)** at 50–600 nM under pH 5.5 in association steps (120 s). The sensor chips were subsequently soaked with dissociation buffer (120 s). **(D)** Comparison of the binding among IgY-Fc mutants (WT, Y363A, and G365A) at 200 nM to FcRY. WT, recombinant quail wild-type IgY-Fc.

To analyze binding properties and stability in more detail, the binding affinities and kinetic parameters (association/dissociation rate) between the mutants and FcRY were determined using a BLI system. At pH 5.5, all mutants bonded to FcRY in a dose-dependent manner (**Figures 5A–C**), but Y363A bonded more strongly to FcRY (**Figure 5D**). At pH 7.4, binding of IgY and its mutants to FcRY markedly decreased (**Supplementary Figures 3B-E**). Their K_D_, *k_a_*, and *k_d_* values were calculated using BLItz-specific software (**Table 1**). The K_D_ of IgY was 275 nM, which was comparable to that obtained previously using surface plasmon resonance (4). G365A showed a greater association rate (2.2-fold) and stronger binding affinity than WT (K_D_; 197 nM of G365A vs. 333 nM of WT). However, Y363A had a lower dissociation rate (approximately 1.5-fold) and stronger binding affinity than WT (K_D_; 65 nM of Y363A vs. 333 nM of WT). These results suggest that G365A binds to FcRY more efficiently than the WT, and that Y363A, once bound to FcRY, hardly ever dissociates.

**Table 1:** K_D_, *k_a_* and *k_d_* values at pH 5.5 derived from BLItz.

IgY or IgY-Fc (concentration; nM)	K_D_ (nM)	*k_a_* (1/Ms)	*k_d_* (1/s)	R^2^
qIgY (100, 200, 300, 400, 600)	275	3.5×10^4^	9.5×10^-3^	0.977
WT (100, 200, 400, 600)	333	1.9×10^4^	6.5×10^-3^	0.982
Y363A (50, 100, 150, 200, 300, 400, 600)	65	2.3×10^4^	1.5×10^-3^	0.999
G365A (100, 200, 300, 400, 600)	197	4.2×10^4^	8.2×10^-3^	0.985

Table includes the binding affinity of qIgY and IgY-Fc mutants to FcRY calculated the sensorgrams in **Figures 5A-C** using BLItz Pro Software.

### Inhibition of FcRY by neutralizing antibodies and its effect on IgY transport into egg yolks in laying birds

**Figure 6:**
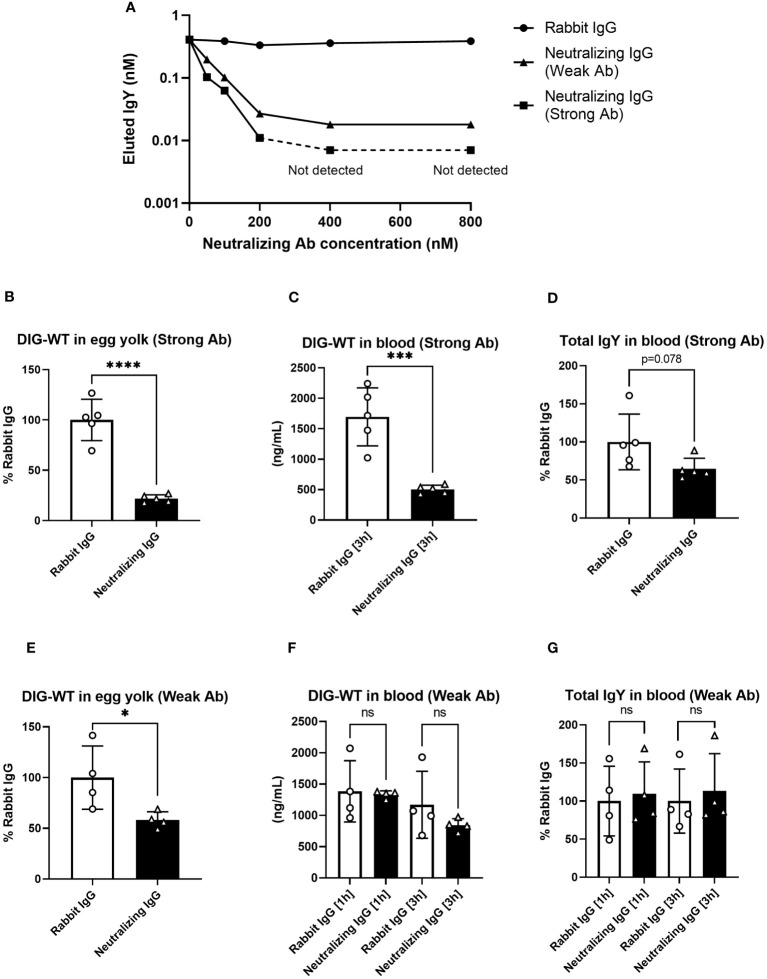
The injection of neutralizing antibodies against FcRY reduces IgY transport into the egg yolks of laying quail. **(A)** Graded amounts of the neutralizing antibodies against FcRY were added to the FcRY-immobilized 96 well plate with IgY (111.1 nM) at pH 6.0. After washing, a basic solution of pH 8.0 was added to liberate the bound IgY from the FcRY. The concentrations of IgY in the collected solutions were determined using ELISA. The values of the strong Ab at 400 nM and 800 nM were not detected using ELISA. **(B–G)** The regularly laying quail (n = 4, 5) were injected intravenously with 1 mg/100 g BW of FcRY-specific neutralizing antibodies (**B–D**, Strong Ab; **E–G**, Weak Ab) and 20 µg/100 g BW of DIG-labeled WT both diluted in 500 μL of saline. All eggs that were laid were collected for 5 d after injections and the concentration of DIG-WT in egg yolks were measured using ELISA **(B, E)**. Blood samples were collected 1- and 3-h post-injection. The concentrations of the injected WT **(C, F)** and total IgY **(D, G)** in serum were determined using ELISA. Vertical bars indicate mean ± standard deviation. Statistically significant p-values were determined using unpaired Student’s *t*-tests: *p < 0.05, ***p < 0.001, ****p < 0.0001. WT, recombinant quail wild-type IgY-Fc.

To obtain direct evidence that FcRY was responsible for IgY transport to the egg yolk, we generated FcRY-specific antibodies. When FcRY antigen was administered to different rabbits, two lots of the antibodies against FcRY with different neutralizing activities were obtained. Both antibodies neutralized IgY binding to FcRY in a dose-dependent manner (**Figure 6A**). The first neutralizing antibody (called Strong Ab) efficiently and strongly inhibited IgY binding compared to the second neutralizing antibody (called Weak Ab).

Next, each neutralizing antibody was intravenously co-administered with DIG-labeled IgY-Fc (WT) tracer to the laying quail. Prior to the experiment, we confirmed both neutralizing antibodies did not interact with IgY by Western blotting and immunohistochemical analyses. In quail treated with Strong Ab, the tracer uptake in the yolks was reduced to approximately 20% of that in the control rabbit IgG (**Figure 6B**). The blood tracer concentration collected 3 h after the administration showed a substantial reduction to about 30% of the control (**Figure 6C**). Similarly, the total IgY concentration in the blood was also reduced to approximately 65% (**Figure 6D**). In quail treated with Weak Ab, the tracer transported to the yolks was also reduced to ~60% (**Figure 6E**). However, no differences were observed in the blood tracer concentrations (**Figure 6F**) or the total blood IgY concentrations (**Figure 6G**). Thus, the inhibition of FcRY by the Weak Ab reduced IgY uptake in egg yolks with minimal impact on blood IgY concentrations. These results indicate that FcRY controls maternal IgY transfer to the yolks in avian species.

## Discussion

The results of this study are the first to demonstrate that avian FcRY plays a key role in IgY transfer from maternal blood circulation to egg yolks in maturing oocytes of laying birds. Our main findings were: 1) the majority of FcRY in ovarian follicles was expressed in capillary endothelial cells of the internal theca layer, 2) binding properties of IgY-Fc mutants/FcRY closely matched the transport ability of the IgY-Fc mutants into the egg yolk, 3) the injection of neutralizing antibodies against FcRY reduced IgY uptake into egg yolk in the hen. These results support the hypothesis that maternal–newly-hatched IgY transfer in the two steps is regulated by a single receptor, FcRY, in avian species. The FcRY-dependent IgY transport system to the egg yolks may be a basic mechanism, broadly applicable to maternal immunity in reptiles as well as avian species.

Analysis of the FcRY/IgY complex structure by cryoelectron microscopy showed that FcRY is in close contact with IgY Cυ4 domain and its surroundings (11). The LYI (362–364 at Cυ3) and HEAL motif (550–553 at Cυ4) within the interface of Cυ3 and Cυ4 domain are conserved well in transportable Igs into egg yolks (20, 21). The substitution of one amino acid residue in the region to other amino acids markedly impacts the uptake of mutants into egg yolks (7, 8). The G365A mutant with a high transport efficiency into egg yolks bonded strongly to FcRY, with much higher association and dissociation rates than WT (**Figures 4**, **5**; **Table 1**). The G365 residue is next to α-helix from S358 to I364, and it constitutes an exposed surface area of IgY-Fc (PDB ID 2W59). Predicted structure of the FcRY/WT complex by AlphaFold2 (22) provided an intermolecular hydrogen bond between the R841 residue in cysteine rich-domain 5 (CTLD5) of FcRY and the G365 residue of WT (**Supplementary Figure 4C** **;** **Supplementary Table 2**). This hydrogen bond was also observed in the FcRY/G365A complex (**Supplementary Figure 4E**). Another docking simulation by HADDOCK (23, 24) predicted that the A365 residue in the G365A mutant formed two hydrogen bonds to the R900 residue in FcRY (**Supplementary Figure 5C** **;** **Supplementary Table 2**), whereas the G365 residue of the WT formed only a single hydrogen bond with the R900 residue in FcRY (**Supplementary Figure 5A**). The results suggest that the G365A mutant strongly binds to FcRY via the formation of additional hydrogen bond. We speculate that, in the maternal body, the G365A mutant efficiently bind to FcRY in capillary endothelial cells, followed by the prompt release of G365A into extravascular ovarian tissues, thus resulting in a marked increase in G365A uptake in egg yolks. Several mutants (G365D and N408A), which are less likely to be transported to egg yolks, showed lower competitive ability against the binding of IgY to FcRY (**Figure 4D**). Variations in binding affinity between the FcRY and IgY-Fc mutant could explain the differences in the mutant uptake into egg yolks of laying birds.

In contrast, Y363A, a mutant with no ability to transport Igs into egg yolks, bonded strongly to FcRY, and its dissociation from FcRY was much slower than that of the WT and G365A mutant. Microscopic observations of Y363A injected into the ovarian follicles indicated its deposition in the vicinity of internal theca layer (**Figure 3C**-iv–vi). These results suggest that Y363A is strongly bound to the FcRY with less dissociation. The Y363 is positioned at the α-helix from S358 to I364, which are located in an exposed loop in close proximity to another loop of the Cυ4 domain (25). Side chain of the Y363 residue forms hydrogen bond with a side chain of the E551 at Cυ4 domain (PDB ID 2W59), and therefore contributes conformational stability of the interface of Cυ3 and Cυ4 domain. In support of this notion, the predicted Y363A mutant structure was dissimilar to that of the WT in terms of the angle between Cυ3 and Cυ4 domain (RMSD value 3.855; **Supplementary Figure 4A**), whereas the G365A mutant structure was nearly identical to that of the WT (RMSD value 0.470; **Supplementary Figure 4B**). Interestingly, the prediction results indicated that the both Y363 and A363 residues did not form any intermolecular hydrogen bonds to FcRY (**Supplementary Figures 4C, D** **;** **Supplementary Table 2**). In addition, the docking simulation by HADDOCK showed that the Y363A mutant bound to FcRY at distinct binding sites from those found in the WT and G365A mutant (**Supplementary Figures 5A-C**). We speculate that substitution of the Y363 to Ala may cause massive conformational change at Cυ3/Cυ4 domain, which might produce strong binding activity to FcRY (**Figure 4B**). However, the competitive ability of the Y363A mutant against IgY-FcRY binding was equivalent to that of the WT (**Figure 4C**). One plausible explanation is that the Y363A mutant gains binding ability to another FcRY region distinct from IgY- and WT-binding region on FcRY. Taken together, the Y363A mutant appears to have complex binding mode to FcRY dissimilar to other IgY-Fc mutants.

**Figure 7:**
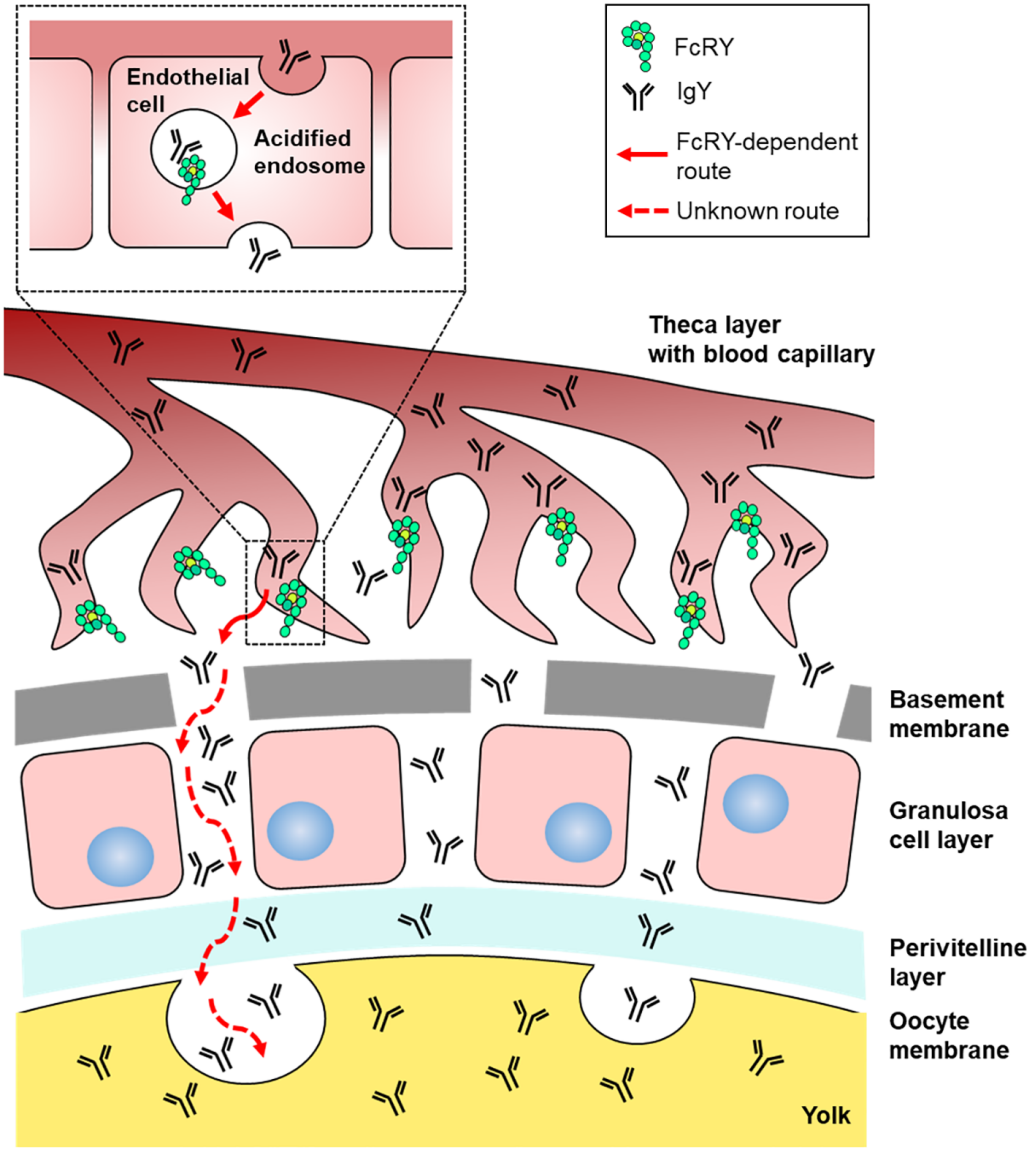
Graphical illustration of the IgY transport pathway in the ovarian follicle of laying birds. FcRY would localize within the acidified endosomes of capillary endothelial cells in the internal theca layer and transport IgY to the extravascular space. Transported IgY from capillary endothelial cells in the theca layer has to pass through several membranes/layers of ovarian follicle. The basement membrane, one of the membranes just inside the theca layer, permits penetration of particles, thus IgY can move across the basement membrane. It is unclear how IgY is transported across other membranes/layers of ovarian follicle (granulosa cell layer, perivitelline layer, and oocyte membrane). Finally, the IgY is incorporated into yolks probably due to endocytosis.

The precise mechanisms by which FcRY transports IgY using a pH-dependent binding property remain unclear. Immunofluorescent assays revealed that FcRY seemed to express intracellularly in capillary endothelial cells in internal theca layer of the ovarian follicle (**Figure 2D**). In mammals, maternal Igs are transported to the neonate via placenta and breast milk (26, 27). FcRn, which is structurally similar to class I major histocompatibility complex (MHC), is responsible for the transport of maternal IgG. The binding characteristic of mammalian FcRn are nearly the same as those of avian FcRY observed here. FcRn binds to the Fc region of IgG at the acidic pH, but dissociates from IgG at the physiological pH (26, 28). The strict pH dependence of the FcRn-IgG interaction is mediated by several amino acid residues in the Cγ2-Cγ3 region of IgG, and one of which is also conserved in cIgY (hIgG LMI251-253, cIgY LYI362-364) (29–33). Mammalian FcRn is localized in early and recycling endosomes of syncytiotrophoblast of the placenta (34, 35), and transports IgG from maternal circulating blood to fetal circulating blood (27, 36). The pH within early (pH ~6.0) and recycling endosomes (pH ~6.5) is mildly acidic (37, 38). It is quite convincing that all IgY-Fc mutants bound strongly to FcRY at pH 5.0–6.0, but not above pH 6.5 (**Figure 4B**). It is therefore possible that FcRY may localize within the endosomes of capillary endothelial cells like mammalian FcRn (**Figure 7**). Transported IgY from capillary endothelial cells in the theca layer has to pass through several membranes/layers of ovarian follicle (39). The basement membrane, one of the membranes just inside the theca layer, permits penetration of particles of < 40 nm (40, 41), thus IgY (< 20 nm) can move across the basement membrane. However, it is unclear how IgY is transported across other membranes/layers of ovarian follicle (granulosa cell layer, perivitelline layer, and oocyte membrane). FcRY at protein level was not detected in these layers except for theca layer (**Figures 2C, D**). Further research is needed to elucidate the mechanism of FcRY-dependent IgY transport in capillary endothelial cells and the subsequent transport pathway of IgY until incorporation in the yolks.

Avian FcRY was first discovered in the yolk sac membrane during embryogenesis (4, 9). The uptake of yolk-deposited IgY through the yolk sac membrane dramatically increases in the last few days before hatching (1), which coordinates enhanced FcRY gene expression in the yolk sac membrane (unpublished data), implying that the yolk-deposited IgY transport to embryonic circulation is a brief event associated with rapid uptake of IgY. In contrast, deposition of yolk protein into ovarian follicles requires relatively long-term period over 2 months (42). Injection study of radio-labeled IgY into laying hens revealed that the injected IgYs were transported into yolks of both the small white ovarian follicles and large yellow ovarian follicles (43). In support of this, FcRY was expressed in both the white and yellow ovarian follicles in the present study (**Figure 2C**). Considering strong FcRY expression in the yolk sac membrane (**Supplementary Figure 1**, **Supplementary Table 1**), IgY-transport efficiency and capability of the second step via the yolk sac membrane would be much higher than those of the first step via the capillary endothelial cells in ovarian follicles.

Another interesting finding was that the injection of neutralizing antibodies in laying quails markedly reduced not only IgY uptake into the egg yolks, but also the IgY concentration in the blood (**Figures 6C, D**). To date, no study has investigated the regulatory mechanism of blood IgY half-life in avian species. Our findings indicate that FcRY is also involved in prolongation of blood IgY half-life. Immunohistochemistry revealed that chicken FcRY is expressed in various cell types, such as the liver sinusoidal endothelial cells and splenic/intestinal/lung lymphocyte-like cells (**Supplementary Table 1**), which is where mammalian FcRn is expressed (44–46). Mammalian macrophages, monocytes, and vascular endothelial cells expressing FcRn are responsible for IgG recycling, which protects intracellular uptake of IgG from lysosomal degradation and transports it back into the bloodstream, increasing the half-life of blood IgG (47, 48). FcRn ablation results in substantially less blood IgG in mice (49). A polarized mammalian epithelial cell line expressing FcRY was shown to endocytose and recycle IgY (12). Thus, avian FcRY and mammalian FcRn are expected to be structurally distinct molecules but are functionally similar to the receptor controlling blood IgY-half life.

In conclusion, this study indicates that avian FcRY plays a major role in maternal blood IgY transfer into egg yolks. We found for the first time that FcRY was expressed in the capillary endothelial cells of ovarian follicles. Avian FcRY contributes to IgY recycling to extend the half-life of IgY in the bloodstream. The main limitation of this study is a lack of evidence that complete ablation of the *FcRY* gene inhibits transport of maternal blood IgY into egg yolks. Production of *FcRY* gene knockout/knockin birds using primordial germ cells and gene editing system would elucidate avian maternal immunity. The *FcRY* gene-modified birds also help find out novel immune functions of FcRY explaining the long half-life of IgY in blood. Finally, it would be possible to provide a new strategy for increasing an amount of IgY in avian egg yolks and enhancing avian immunity by controlling the FcRY/IgY interaction. A careful consideration of the immune abnormality caused by the modified FcRY/IgY interaction is necessary to achieve that aim.

## Data availability statement

The original contributions presented in the study are included in the article/**Supplementary Material**, further inquiries can be directed to the corresponding author.

## Ethics statement

The animal study was approved by Nagoya University Policy on Animal Care and Use. The study was conducted in accordance with the local legislation and institutional requirements.

## Author contributions

MO: Investigation, Writing – original draft. RS: Investigation, Writing – review & editing. KoI: Investigation, Writing – review & editing. KD: Investigation, Writing – review & editing. FT: Investigation, Writing – review & editing. KO: Methodology, Writing – review & editing. TK: Methodology, Writing – review & editing. SM: Writing – review & editing. KeI: Methodology, Writing – review & editing. TY: Methodology, Writing – review & editing. KF: Writing – review & editing. MK: Writing – review & editing. FH: Writing – review & editing. AM: Project administration, Writing – original draft.
