# Supplementary material for: FcRY is a key molecule controlling maternal blood IgY transfer to yolks during egg development in avian species
Source: Front Immunol. 2024 Feb 29;15:1305587. doi: 10.3389/fimmu.2024.1305587 (PMC10938909; doi:10.3389/fimmu.2024.1305587)
Supplement: Supplementary file 1 [file DataSheet_1.docx]

In
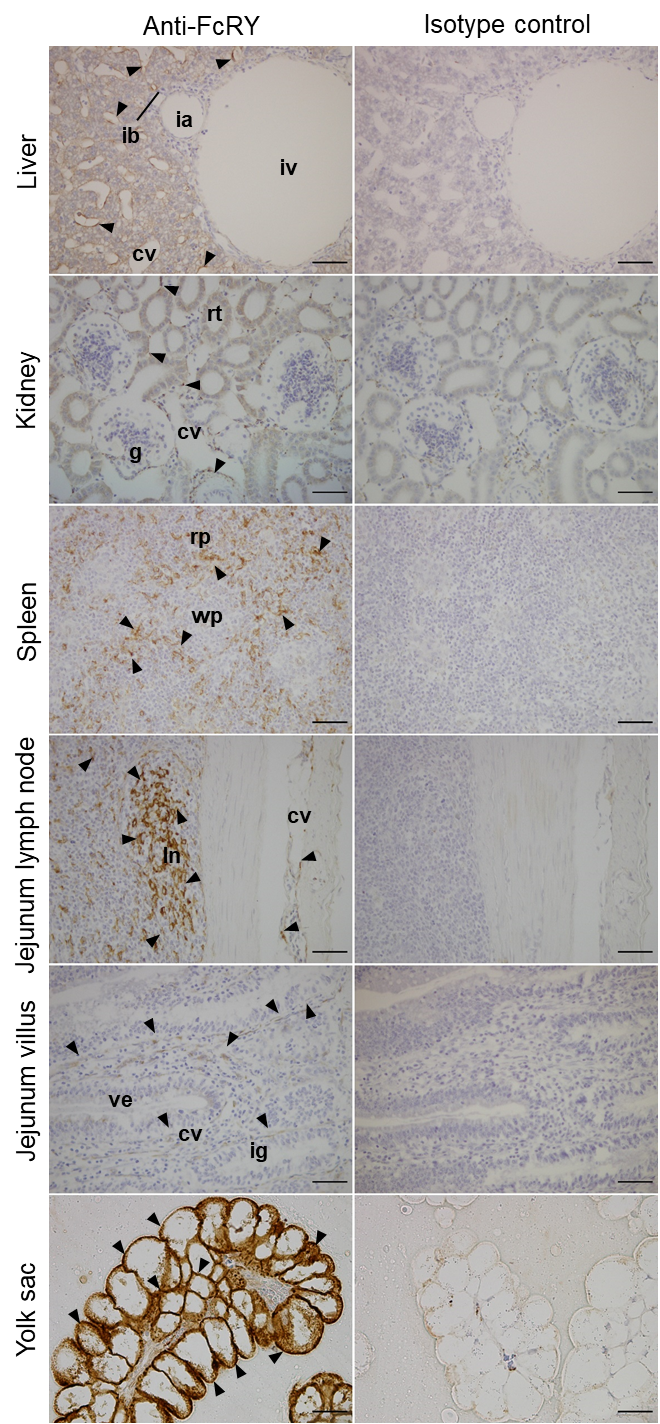


**Supplemental Figure 1.** FcRY is localized in various organs of chicken. FcRY signal localizations were verified with anti-FcRY antibody (left) or nonspecific antibody (right) by immunohistochemistry (original magnification, x40). Scale bar, 40 μm. cv, capillary vessel; g, glomerulus; ia, interlobular artery; ib, interlobular bile duct; ig, intestinal gland; iv, interlobular vein; ln, lymph node; rp, red pulp; rt, renal tubule; ve, villi epithelium; wp, white pulp

**Supplemental Table 1.** Localization of FcRY in chicken organs by immunohistochemistry

| Organ | Tissues / Cells | FcRY abundance |
| --- | --- | --- |
| Ovarian follicle | Theca interna / endothelial cells | +++ (basolateral) |
|  | Granulosa cells | - |
|  | Theca externa / endothelial cells | + |
| Yolk sac | Yolk sac membrane / epithelium cells | ++++ (surface) |
| Liver | Sinusoid / endothelial cells | +++ |
|  | Hepatic triad / endothelial cells | - |
| Kidney | Renal proximal tubule / epithelial cells | + |
| Spleen | White pulp | - |
|  | Red pulp / lymphocytes | +++ |
|  | Red pulp / endothelial cells | ++ |
| Thymus | Vein / endothelial cells | + |
|  | Lymphocyte like cells | + |
| Jejunum | Lymph node / lymphocytes | +++ |
|  | Villus / endothelial like cells | + |
|  | Mucosal / epithelium cells | - |
| Lung | Capillary / endothelial cells | - |
|  | Lymphocyte like cells | ++ |
|  | Alveolar / epithelial like cell | + |
| Aorta | - | - |
| Carotid artery | - | - |
| Jugular vein | - | - |

Staining intensity and abundance indicated either with + (mild intensity / expressed only in a subset of cells of this type), ++ (medium intensity), +++ (strong intensity / expressed in most cells of this type), ++++ (very strong intensity) or − (no staining in this cell type).


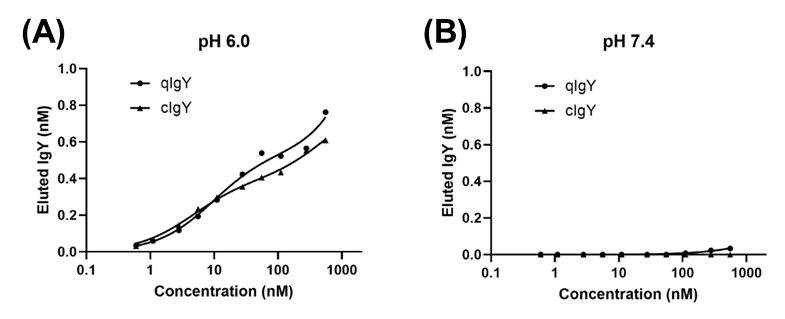


**Supplemental Figure 2.** Both chicken and quail IgYs bonded to FcRY at pH 6.0 but not bonded at pH 7.4 at all. (**A and B**) Copper-coated 96 well plates were incubated with secretory FcRY (10 μg/ml). The plates were then incubated with the chicken or quail IgY (0.56-556 nM; n = 1). After washing, basic solution with pH 8.0 was added to liberate bound Igs from the FcRY. The concentrations of IgYs in the collected solutions were determined by ELISA.


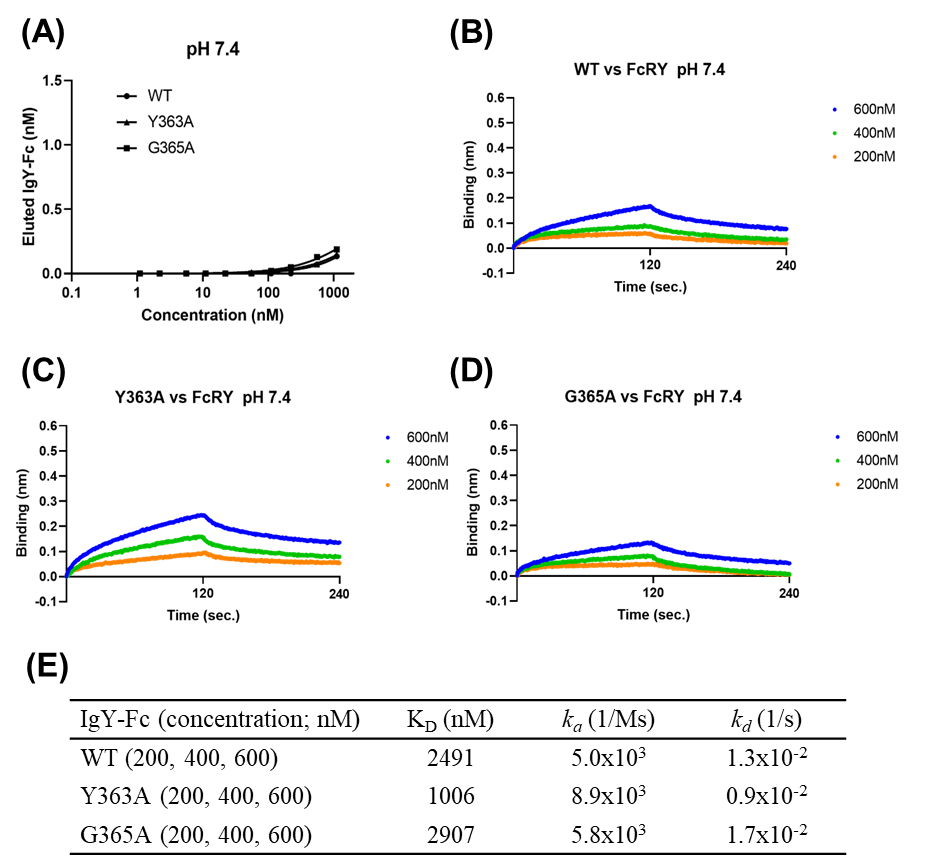


**Supplemental Figure 3.** None of the IgY-Fc mutants bonded to FcRY at pH 7.4. (**A**) Copper-coated 96 well plates were incubated with secretory FcRY (10 μg/ml). The plates were then incubated with the WT, G365A and Y363A (1.1-1,111 nM). After washing, basic solution with pH 8.0 was added to liberate bound Igs from the FcRY. The concentrations of IgY-Fc mutants in the collected solutions were determined by ELISA. (**B**-**D**) FcRY (400 nM) was immobilized on the sensor chip. The sensor chips were dipped into WT (B), Y363A (C) or G365A (D; 200-600 nM at pH 7.4, respectively) solutions in association step (120 s). The sensor chips were subsequently soaked with dissociation buffer (120 s). (**E**) Table includes the binding affinity of IgY-Fc mutants to FcRY at pH 7.4 calculated using BLItz Pro Software.

**
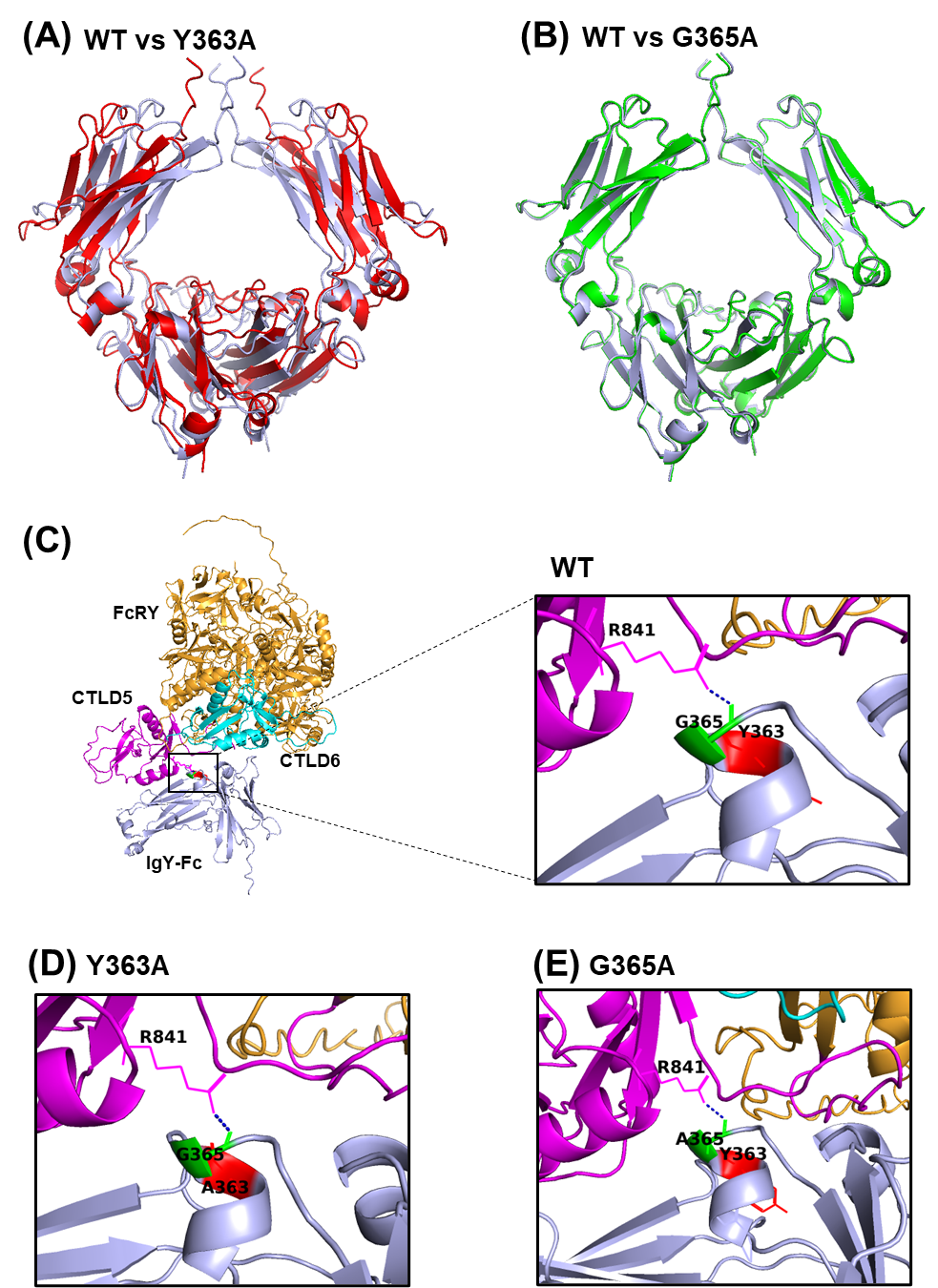
**

**Supplemental Figure 4.** Predicted structures of the IgY-Fcs (WT, Y363A, G365A) and the FcRY/IgY-Fc complexes by AlphaFold2. The FcRY (residues 24 to 1398) and IgY-Fc (residues 342 to 568) structures were modeled by AlphaFold2 using ColabFold interface (ver. 1.5.5). The chicken IgY-Fc structure (PDB 2W59) and the chain A of chicken IgY-Fc structure (PDB 2W59_A) were uploaded as a template of dimeric IgY-Fc and monomeric IgY-Fc, respectively. The combined structural modeling was performed with AlphaFold multimer v2 predictions with 3 iterances of model recycling. The predicted structures were ranked by the predicted local distance test (plDDT) scores and by the Predicted Aligned Error (PAE), and the rank1 structures were shown in this figure. (**A and B**) Comparisons of the dimeric IgY-Fc structures between the WT (light purple) and the Y363A (red) (A), and between the WT and the G365A (green) (B). (**C**) The structure of FcRY/WT complex. The FcRY amino acid residues are colored by magenta (CTLD5), cyan (CTLD6) and orange (others), and the IgY-Fc amino acid residues are colored by light purple. The zoom-in view of the residues near the Y363 (red) and the G365 (green) was shown in the square on the right. The G365 forms a hydrogen bond with the R841 of FcRY (blue-dashed line). (**D**) The zoom-in view of the residues near the A363 and the G365 of the FcRY/Y363A complex. (**E**) The zoom-in view of the residues near the Y363 and the A365 of the FcRY/G365A complex.


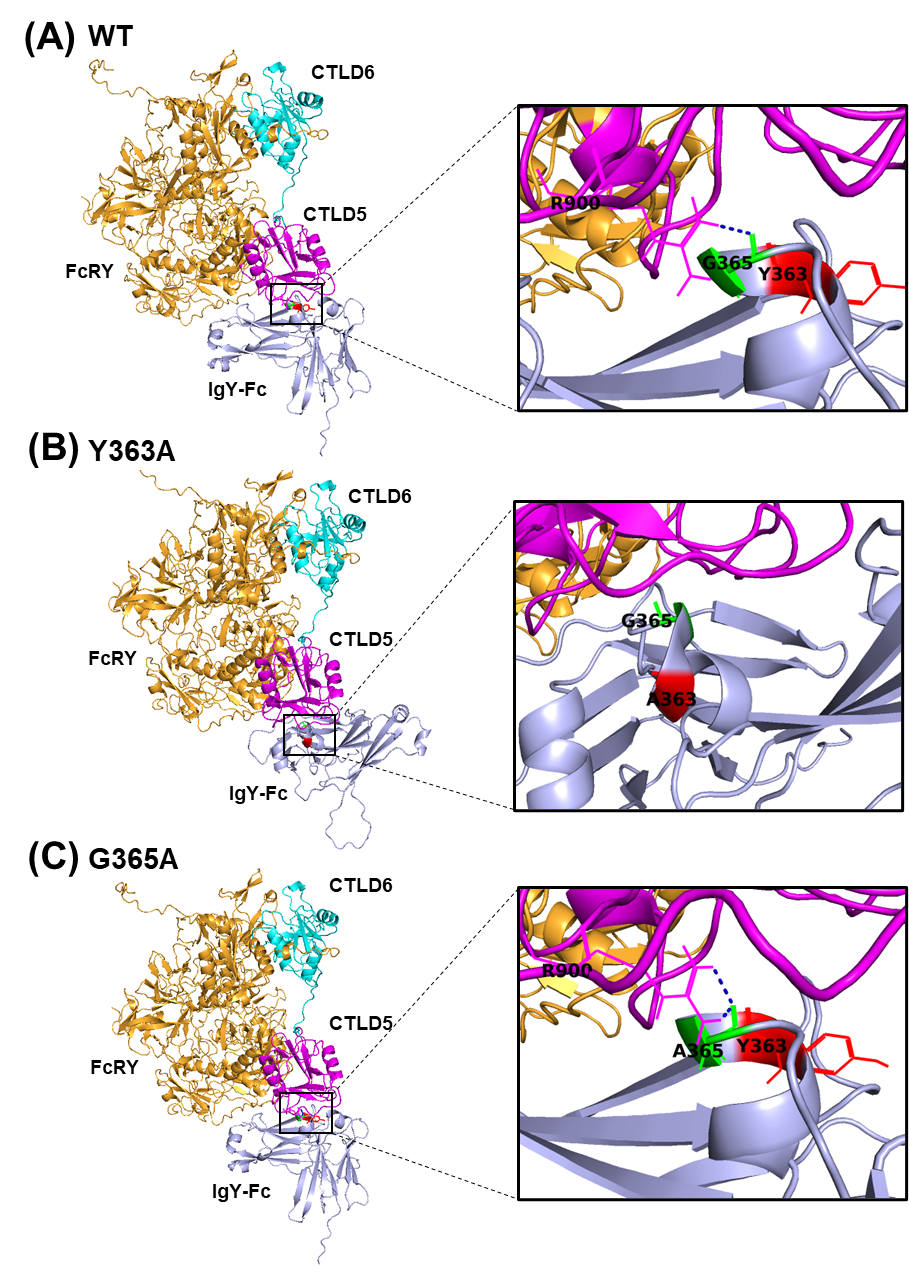


**Supplemental Figure 5.** Molecular docking simulation of the FcRY/IgY-Fc complex by HADDOCK. (**A-C**) For each structure in the dataset, FcRY structure and IgY-Fc structure were predicted by AlphaFold2 using ColabFold interface (ver. 1.5.5). The protein-protein docking simulation were performed using HADDOCK (ver. 2.4), and the structure with the least HADDOCK score were adopted in this study. The FcRY/WT (A), the FcRY/Y363A (B) and the FcRY/G365A (C) complexes are shown on the left, and the zoom-in views of the residues near the Y363 (or A363; red) and the G365 (or A365; green) were shown in the square on the right. The FcRY amino acid residues are colored by magenta (CTLD5), cyan (CTLD6) and orange (others), and the IgY-Fc amino acid residues are colored by light purple. The G365 of WT and the A365 of G365A form hydrogen bonds with the R900 of FcRY (blue-dashed line).

S**upplemental Table 2.** Predicted interaction residues between FcRY and IgY-Fcs (WT, Y363A, G365A)

| Software | IgY-Fc | |  | FcRY |
| --- | --- | --- | --- | --- |
|  | Name | Residue |  | Residue |
| ColabFold | WT | Y363 |  | - |
|  | Y363A | A363 |  | - |
|  | G365A | Y363 |  | - |
|  | WT | G365 |  | R841 (N-H 2) |
|  | Y363A | G365 |  | R841 (N-H 2) |
|  | G365A | A365 |  | R841 (N-H 2) |
| HADDOCK | WT | Y363 |  | - |
|  | Y363A | A363 |  | - |
|  | G365A | Y363 |  | - |
|  | WT | G365 |  | R900 (H 11) |
|  | Y363A | G365 |  | - |
|  | G365A | A365 |  | R900 (H 11) R900 (H 21) |

Table shows the amino acids residues in FcRY that form hydrogen bonds with the 363 or 365 residues in IgY-Fc. -, there is no hydrogen bond in FcRY amino acid residues. Position of molecules forming hydrogen bond with IgY-Fc is shown in parentheses.
